# Magnetic Particle / Magnetic Resonance Imaging: *In-Vitro* MPI-Guided Real Time Catheter Tracking and 4D Angioplasty Using a Road Map and Blood Pool Tracer Approach

**DOI:** 10.1371/journal.pone.0156899

**Published:** 2016-06-01

**Authors:** Johannes Salamon, Martin Hofmann, Caroline Jung, Michael Gerhard Kaul, Franziska Werner, Kolja Them, Rudolph Reimer, Peter Nielsen, Annika vom Scheidt, Gerhard Adam, Tobias Knopp, Harald Ittrich

**Affiliations:** 1 Department of Diagnostic and Interventional Radiology and Nuclear Medicine, University Medical Center Hamburg-Eppendorf, Hamburg, Germany; 2 Section for Biomedical Imaging, University Medical Center Hamburg-Eppendorf, Hamburg, Germany; 3 Institute for Biomedical Imaging, Hamburg University of Technology, Hamburg, Germany; 4 Microscopy and Image Analysis, Heinrich Pette Institute, Leibniz Institute for Experimental Virology, Hamburg, Germany; 5 Department of Biochemistry and Molecular Cell Biology, University Medical Center Hamburg-Eppendorf, Hamburg, Germany; 6 Department of Osteology and Biomechanics, University Medical Center Hamburg Eppendorf, Hamburg, Germany; Institute for Frontier Medical Sciences, Kyoto University, JAPAN

## Abstract

**Purpose:**

*In-vitro* evaluation of the feasibility of 4D real time tracking of endovascular devices and stenosis treatment with a magnetic particle imaging (MPI) / magnetic resonance imaging (MRI) road map approach and an MPI-guided approach using a blood pool tracer.

**Materials and Methods:**

A guide wire and angioplasty-catheter were labeled with a thin layer of magnetic lacquer. For real time MPI a custom made software framework was developed. A stenotic vessel phantom filled with saline or superparamagnetic iron oxide nanoparticles (MM4) was equipped with bimodal fiducial markers for co-registration in preclinical 7T MRI and MPI. *In-vitro* angioplasty was performed inflating the balloon with saline or MM4. MPI data were acquired using a field of view of 37.3×37.3×18.6 mm^3^ and a frame rate of 46 volumes/sec. Analysis of the magnetic lacquer-marks on the devices were performed with electron microscopy, atomic absorption spectrometry and micro-computed tomography.

**Results:**

Magnetic marks allowed for MPI/MRI guidance of interventional devices. Bimodal fiducial markers enable MPI/MRI image fusion for MRI based roadmapping. MRI roadmapping and the blood pool tracer approach facilitate MPI real time monitoring of *in-vitro* angioplasty. Successful angioplasty was verified with MPI and MRI. Magnetic marks consist of micrometer sized ferromagnetic plates mainly composed of iron and iron oxide.

**Conclusions:**

4D real time MP imaging, tracking and guiding of endovascular instruments and *in-vitro* angioplasty is feasible. In addition to an approach that requires a blood pool tracer, MRI based roadmapping might emerge as a promising tool for radiation free 4D MPI-guided interventions.

## Introduction

Magnetic particle imaging (MPI) is a new imaging modality using superparamagnetic iron oxide particles (SPIOs) as tracer substance [1]. This new radiation-free tomographic imaging method provides fast, background-free, sensitive, directly quantifiable 4 dimensional (4D) information about the spatial distribution of SPIOs at high temporal resolution (milliseconds), spatial resolution (<1 mm), and sensitivity (μmol) [2]. Initial experimental studies demonstrate feasibility in living organisms [3]. Moreover, with optimization of the SPIOs, hardware and software equipment (e.g. reconstruction algorithms), this imaging technology has the potential to detect nano- or picomolar concentrations of SPIOs [4] or to generate 4D images with high temporal resolution, making the modality interesting for molecular imaging and interventional applications. Using the currently available SPIOs (often ferucarbotran (Resovist^®^, Bayer HealthCare AG, Leverkusen, Germany)), a comparable spectrum of applications like in MRI or fluoroscopy is imaginable combined with high sensitivity, good spatial and high temporal resolution. Potential MPI utilizations include cardiovascular applications (angiographies, cardiac vitality evaluation, tissue perfusion, plaque labeling, endovascular interventions, detection of bleeding sources) or applications in tumour, molecular, and cellular imaging (passive and active targeting, molecular therapies, cellular labeling and cell monitoring) [5]. For the broad sector of potential interventional applications, as therapeutic endovascular or thermoablative approaches, Haegele et al. demonstrated promising results in initial experimental studies [6], although potential heating of the material has to be considered [7] and labelling of endovascular devices has to be optimized. For *in-vivo* MPI of interventional instruments currently two strategies are pursued. First, the vessel lumen can be enhanced with long circulation MPI tracers (blood pool SPIOs) [8] or in principle with SPIOs labelled blood cells, e.g. erythrocytes [9, 10]. Second, an anatomical co-registration using a second imaging technique can be realized with image fusion techniques. In this context computed tomography (CT) or magnetic resonance imaging (MRI) data sets would be ideal for the anatomical overlay of the MPI signal.

Using both imaging approaches we designed an MPI-MRI experiment to answer the following questions:

1. Is MPI of magnetically labelled endovascular interventional devices (guide wires, angioplasty balloons) feasible on a preclinical MPI scanner?
2. Does MPI enable real time four dimensional (4D) tracking and guidance of magnetically labelled interventional devices?
3. Is an *in-vitro* angioplasty feasible using SPIOs as a tracer for MPI guidance?
4. Can MPI-MRI roadmapping be realized using bimodal fiducial markers?
5. Does the MPI-MRI overlay approach allow for imaging of a therapeutic angioplasty in an *in-vitro* vessel phantom with a stenosis?

## Materials and Methods

### Magnetic lacquer analysis

For environmental scanning electron microscopy (ESEM), performed and analyzed by R.R. a drop of lacquer was air dried on an epoxy block and trimmed with a diamond knife in a Leica UC7 microtome (Wetzlar, Germany). Imaging was performed with a solid state BSE detector mounted below the pole piece in a Philips XL30ESEM in H_2_O-Mode at 200 Pa, EHT of 15–20 kV and Spot 6. For exact measurement of the iron content atomic absorption spectroscopy (AAS) of previously air dried lacquer using a Perkin Elmer 2100 analyzer (Perkin Elmer, Norwalk, CT, USA) was conducted.

### Endovascular devices and magnetic labelling

The last 5 mm of the guidewires’ tip were dip coated with a magnetic lacquer. PTA balloon catheters were labelled at both sides of the balloon by a manual 360° turn on the lacquer brush. For instrument details see Table 1. The thickness of the lacquer on the instruments was determined by micro-computed tomography (CT) (Skyscan 1272, Bruker, Belgium). Images were obtained at 40 kV and 200 μA at an isotropic resolution of 1.5 μm for the balloon catheter and at 60 kV, 166 μA and 3 μm with a 0.25 mm Al filter for the guidewire. Images were evaluated using the micro-CT programs CTAnalyzer and DataViewer (Bruker, Belgium).

**Table 1:** Endovascular interventional devices.

Device	Brand name	Manufacturer	Wire/Shaft diameter (mm, Inch)	Balloon diameter (mm)	Balloon length (mm)
Guidewire	Radifocus^™^ M Standard type	Terumo, Somerset, USA	0.89, 35/1000		
Angioplasty catheter	Armada 35	Abbott Vascular, Santa Clara, USA	1.73, 68/100	6.0	20

Summary of the used endovascular devices.

### Magnetic Resonance Imaging (MRI) of vessel phantoms

MRI scans were performed using a preclinical 7T MR Scanner (Clinscan 70/30 Bruker Biospin, Ettlingen, Germany) with a transmit-receive body coil (inner diameter: 6.7 cm). The MRI protocol for imaging the vessel phantom consisted of a 2D PD-weighted turbo spin echo sequence. See Table 2 for detailed information on MRI sequence parameters.

**Table 2:** MR imaging parameters.

Sequence	TR (ms)	TE (ms)	FoV (mm)	Matrix	EVV (mm)^3^	SL (mm)	NSA (n)	TA (min.)
2D PD-weighted TSE coronal / sagittal	3500	7	38	192×192	0.158×0.198×0.80	0.8	3	4:10

TR: repetition time; TE: echo time; FoV: field of view; EVV: effective voxel volume; FA: flip angle; SL: slice thickness; NSA: number of acquisitions, TA: total acquisition time

### Magnetic Particle Imaging (MPI)—image acquisition and 4D real time reconstruction

All images were acquired using a commercial preclinical MPI system (Bruker Biospin, Ettlingen, Germany). The selection field gradient was 1.5 T/m, drive fields were applied with an amplitude of 14 mT, defining the size of the measurement field to be 37.3×37.3×18.6 mm³ (length × width × height). All reconstructions were performed using a system function approach (separate calibration scans for each tracer material and gradient setting). Therefore a voxel shaped calibration sample of 2×2×1 mm^3^ was moved to 25×25×25 positions covering a volume of 50×50×15 mm^3^ containing the measurement field. For real time reconstruction and image display a custom made software was developed using the general purpose programming language Julia [11]. The software follows an iterative approach as outlined in [12] and runs parallel to the data acquisition, monitoring the raw measurement data to reconstruct the latest frames. See Table 3 for detailed information on MPI scanning and reconstruction parameters.

**Table 3:** MP imaging and reconstruction parameters.

**MPI Lissajous measurement**	**TR (ms)**	**DF FoV (mm)**		**DF offset (mm)**^**3**^	**NSA (n)**	**TA (min.)**
Device Tracking	21,5	37.3×37.3×18.6		0×0×0	4000	1:26
Fiducial Positioning	21,5	37.3×7.3×18.6		15×0×-10	1000	0:23
Angioplasty	21,5	37.3×37.3×18.6		0×0×0	20000	7:10
**System function**	**VS (mm)**	**FoV (mm)**		**Image size (n)**^**3**^	**MPI tracer**	
SFR	2×2×1	50×50×25		25×25×25	Resovist^®^	
SFL	2×2×1	50×50×25		25×25×25	magnetic lacquer	
**Reconstruction during**	**SF**	**SNR (n)**	**NA (n)**	**Solver**	**I (n)**	**L**
Device Tracking	SFL	5	25	Kaczmarz	5	0.001
Fiducial Positioning	SFR	5	25	Kaczmarz	5	0.01
Angioplasty	SFR	5	25	Kaczmarz	5	0.001

TR: repetition time; DF FoV: drive field field of view; DF offset: drive field offset; NSA: number of acquisitions, TA: total acquisition time; VS: voxel size; FoV: system function field of view; SF: reconstruction system function; SNR: signal-to-noise threshold for frequency selection (SF based); NA: number of averages; I: Iterations; L: normalized Tikhonov-regularization-parameter

### Experimental setup

#### 4D real time tracking of interventional devices

**Fig 1:**
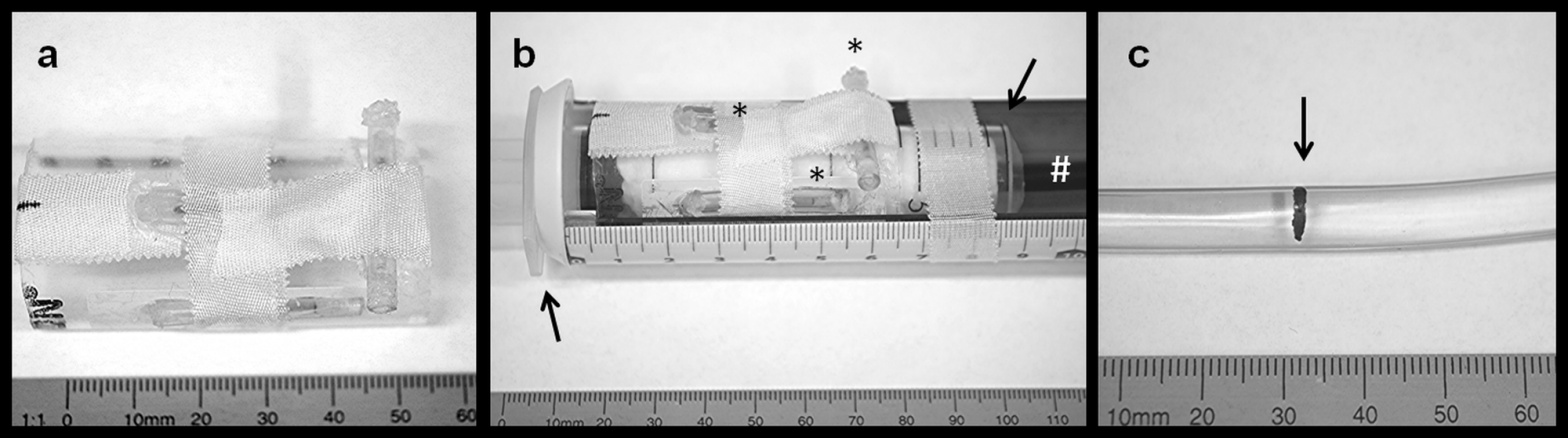
Fiducial and vessel phantoms. **(a)** Photographs of the fiducial phantom with three bimodal MPI-MRI fiducials. Two fiducials are aligned in a T-shape and the last is diagonal below. **(b)** Experimental setup with the vessel phantom (**#**) embedded in a 20 ml syringe (arrows) with three externally fixed fiducials (asterix) placed on the MPI-MRI bench. (**c**) Photograph of the vessel phantom used in the MPI tracking experiment (arrow indicates the lacquer mark).

A thin 2.5 mm wide magnetic lacquer-mark crossway on the vessel phantom indicated the target area as shown in Fig 1c. The phantom was fixed on a MPI/MRI compatible bench (MINERVE, Esternay, France) as shown in Fig 1b. A continuous MPI scan (1:26 min. total scan duration) with real time reconstruction was carried out in two steps: first, the transverse lacquer mark on the vessel phantom was placed in the center of the field of view. Second, positioning the guide wire and coaxially over the wire the balloon catheter within the target area was tried.

#### Angioplasty using MPI/MRI roadmapping and the blood pool tracer approach

A vessel phantom was built using a polyvinyl chloride tube (inner diameter: 4 mm; outer diameter: 6 mm). The tube was sealed watertight and filled with a solution of gadopentetate-dimeglumin (Gd-DTPA (Magnograf^™^, Marotrast, Jena, Germany) containing 0.5 mmol/ml) and 0.9% saline (1:666) or with MM4 (Ferudextran, TOPASS GmbH, Berlin, Germany) (2.8 mg Fe/ml). Stenosis was realized by a ligature (silk suture material). The stenotic vessel phantom, equipped with fiducial markers was fixed on the MPI/MRI bench (see Fig 1a and 1b). MRI scans were performed to quantify and locate the stenosis relative to the fiducial markers. Due to the limited field of view and for joint reconstruction, two MPI scans were carried out: first, the field of view was shifted (10 mm higher and 15 mm to the backside) and the fiducial setup placed within the field of view (23 sec total scan duration). Second, the field of view was shifted back to the region of the stenosis and a dynamic scan (7:10 min total scan duration) (2 frames/sec) was started and reconstructed in real time for device placement and angioplasty by inflating the balloon with a stock solution of MM4 within the saline/Gd-DTPA filled phantom or inflating with saline within a MM4 filled phantom. MRI scans were performed to evaluate the success of angioplasty.

### MPI / MRI image analysis and fusion

Two radiologist and two physicists conducted the image assessment. For visualization and analysis of dynamic image information as well as for quantification of the stenosis additional image processing software (ImageJ, NIH, MD, USA) was used. For road map planning, the 3D MPI volume data with 10 averages of the time frame were fused with a stack of 2D MRI images in sagittal orientation. Image fusion with a rigid transformation was done with the custom software.

## Results

### Magnetic lacquer analysis

**Fig 2:**
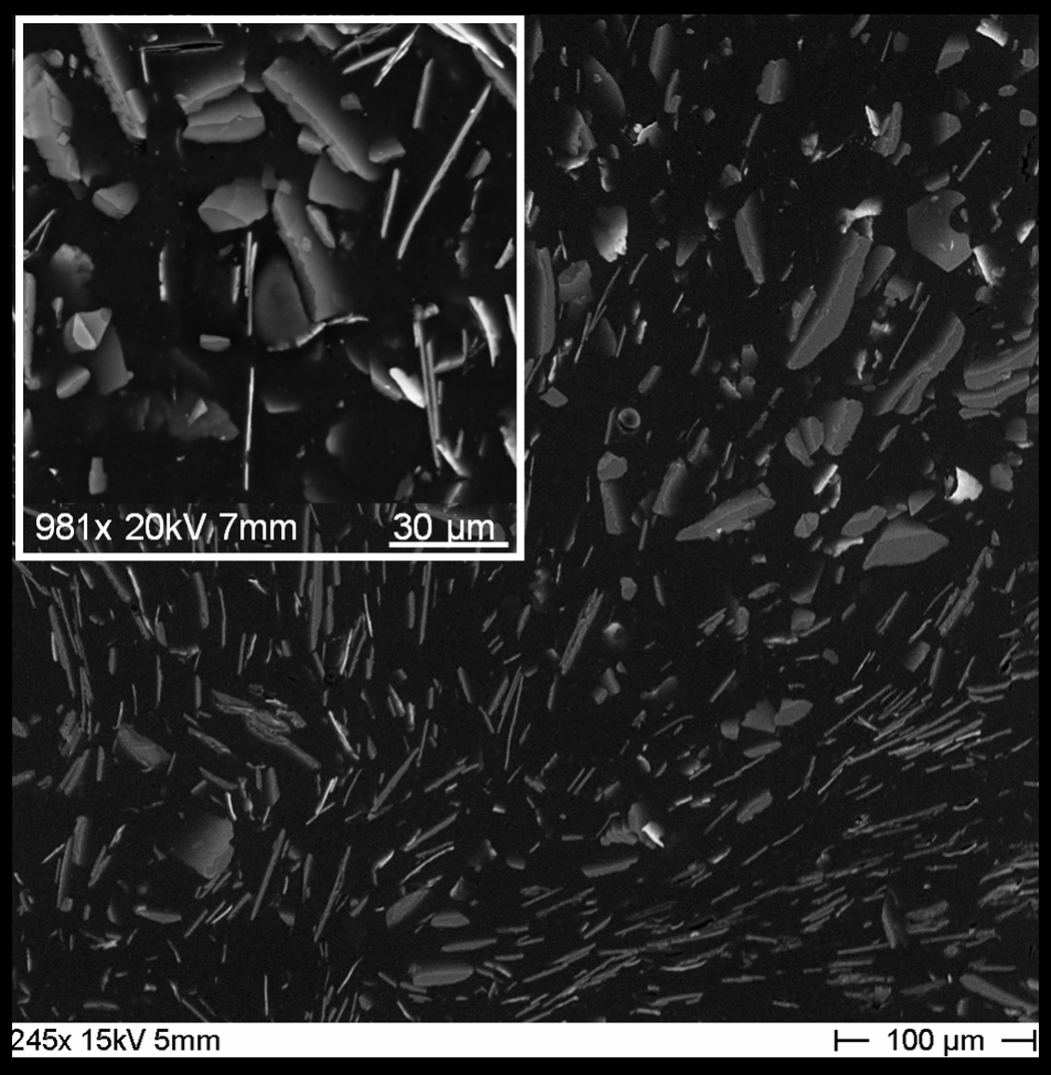
Ultrastructure of the magnetic lacquer. ESEM image of the air dried magnetic lacquer demonstrating highly disperse, ultrathin, plated-like particles from approximately 0.5 to 90 μm in size.

**Fig 3:**
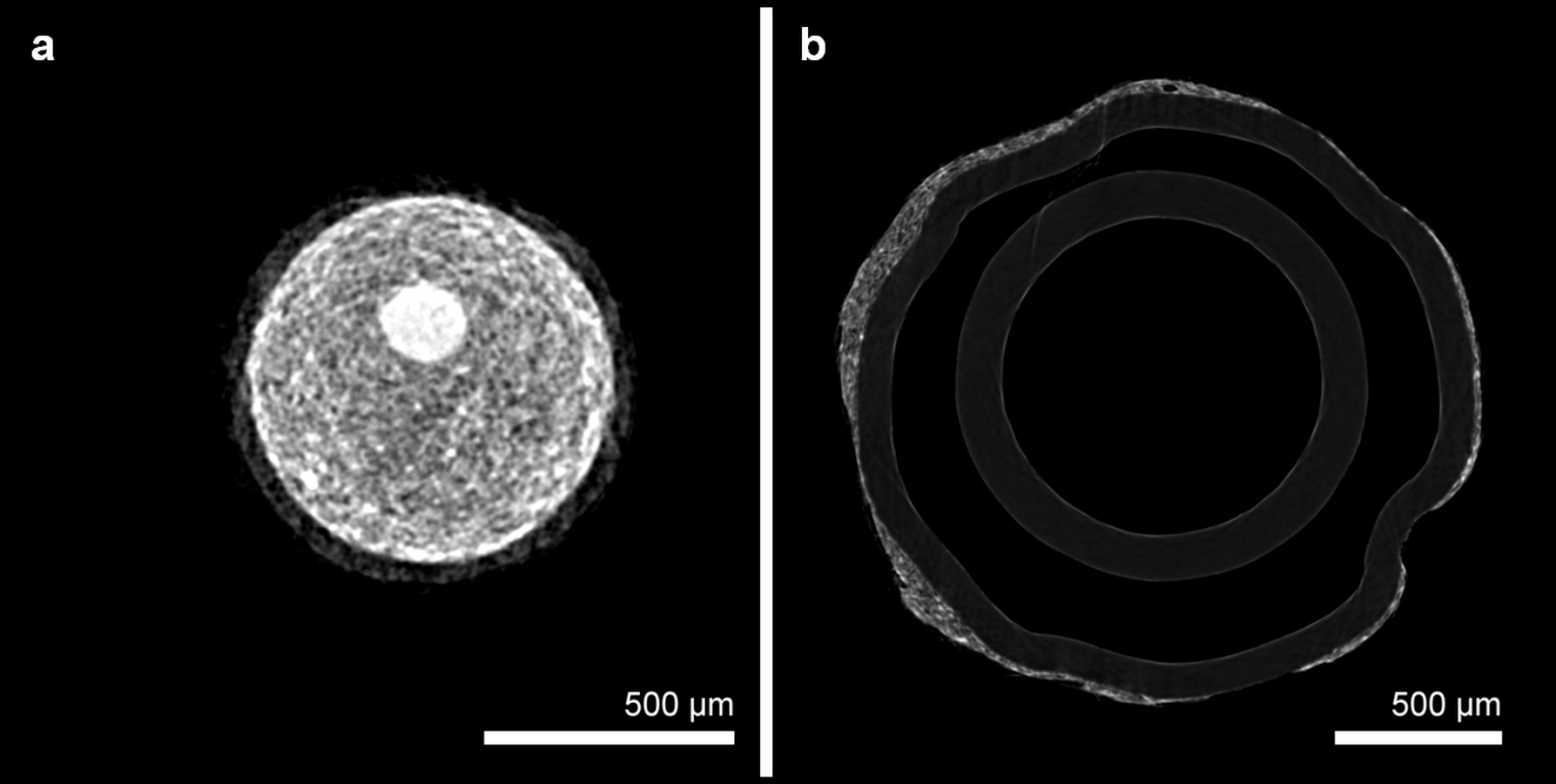
Micro-CT of labelled guidewire and balloon catheter. Micro-CT imaging demonstrates the magnetic lacquer-markings at the tip of the guidewire (**a**) with a maximum thickness of 100μm and adjacent to the balloon of the angioplasty catheter (**b**) with a maximum thickness of 120μm.

ESEM results identified highly disperse thin plates mainly composed of iron and iron oxide with a range in size of 0.5 to approximately 90 μm as the origin of the magnetic properties of the used magnetic lacquer (Fig 2). As depicted by micro-CT the solid layer of magnetic lacquer was considerably homogeneous (Fig 3). By dip coating, the layer on the Radiofocus^FM^ guidewire had a maximum thickness of 100 μm, whereas the PTA balloon catheter showed a layer of maximum thickness of 120 μm (Fig 3). AAS revealed an iron content of 10.6 mg/ml.

### Real time MPI for tracking and positioning of interventional instruments

**Fig 4:**
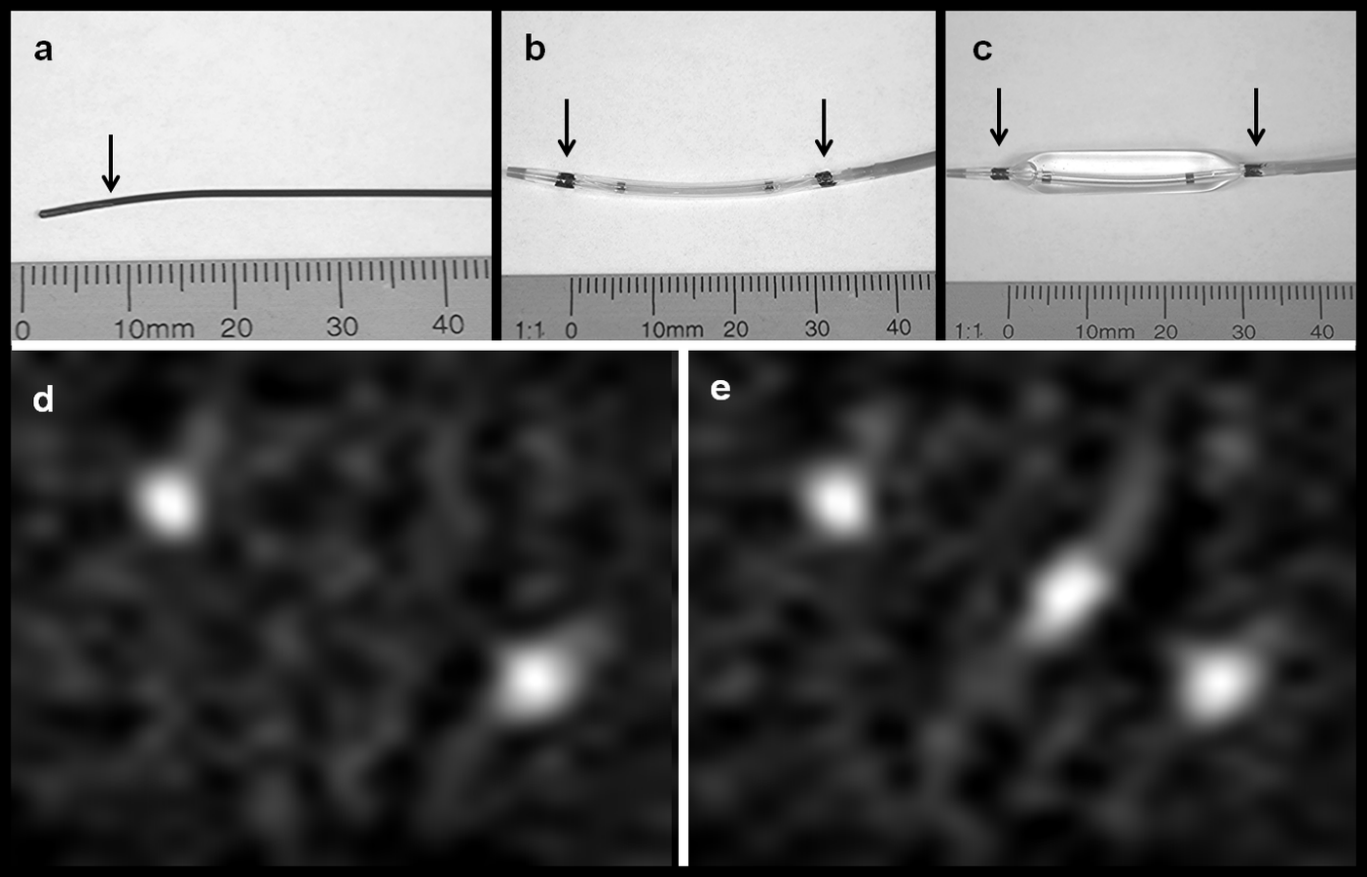
Photographs and MPI of guidewire and PTA balloon catheter. **(a)** Photography of the magnetically labeled Radiofocus^FM^ guidewire (arrow indicates the end thin lacquer-labeling of the tip). **(b)** Photography of the deflated and **(c)** with saline inflated balloon catheter with magnetic markers at both balloon ends (arrows). **(d)** MPI-maximum intensity projection (MIP) in z-projection of the balloon catheter showing the two marks as bright spots. (**e**) MPI-MIP in z-projection of the balloon catheter coaxially placed on the guide wire with the guidewire tip at its center.

Using the described magnetic lacquer marks shown in Fig 4a–4c and the dedicated magnetic lacquer system function, the guidewire and PTA catheter generated a well-defined and strong MPI signal as shown in Fig 4d and 4e. For a most central placement within the field of view, defined by the MPI scanning protocol as specified in Table 3, the PTA balloon catheter and coaxially inserted guidewire were placed slantwise. By choosing lacquer marks of similar size, both marks on the PTA catheter and the mark on the tip of the guidewire have a similar intensity. It was possible to image the tip of the guidewire placed between the two, 3 cm distant marks of the catheter in real time. To demonstrate the low MPI background noise, Fig 4d and 4e are displayed without optimizing the window level. In this setting, there was no dissolving or flaking of the marks.

**Fig 5:**
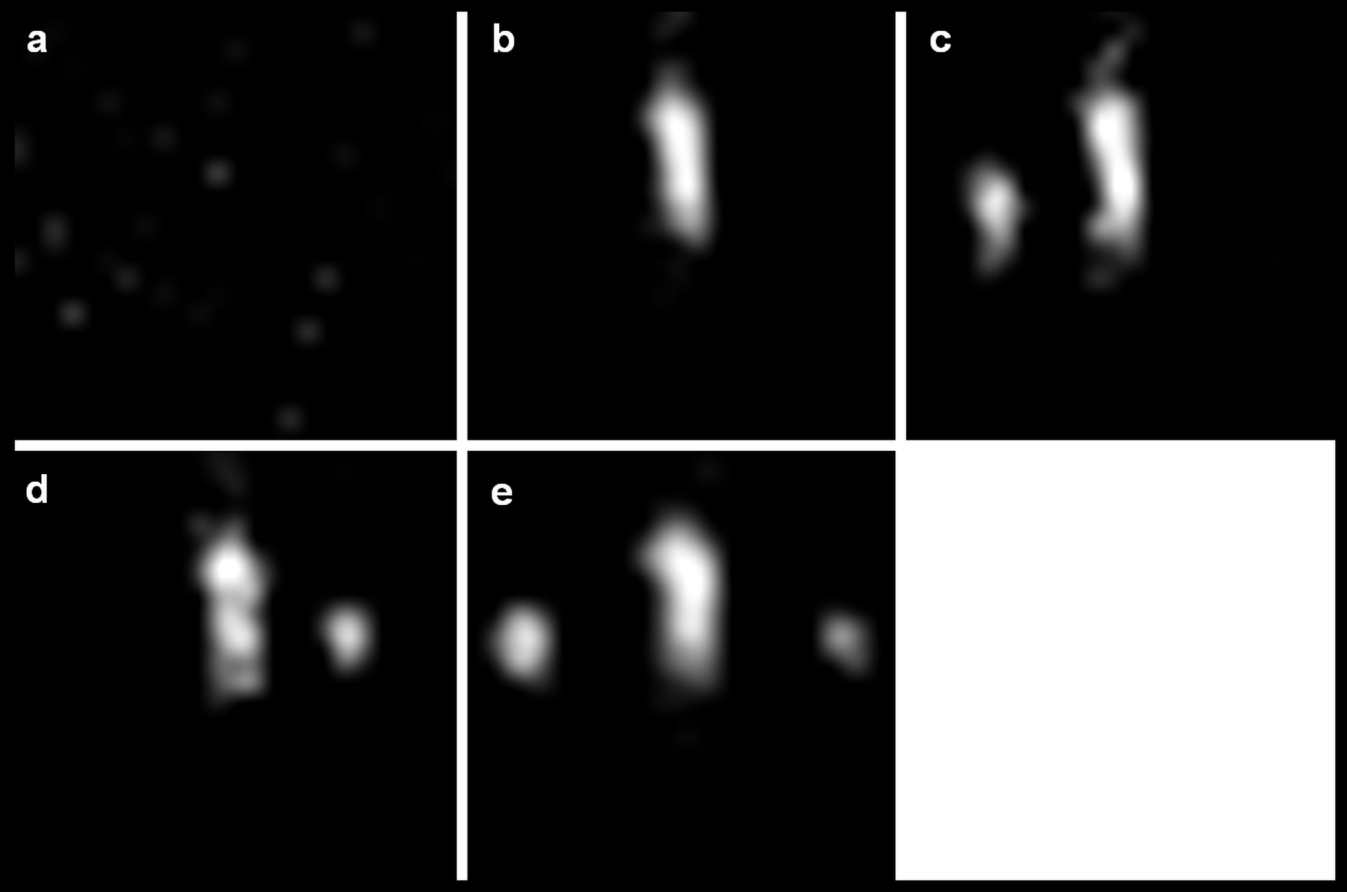
MPI of the positioning procedure. MPI maximum intensity projections (MIP) in z-projection of (**a**) the field of view before placing the vessel phantom, (**b**) magnetic lacquer mark (landing zone) of the vessel phantom (compare Fig 1c), (**c**) the guide wire moved towards the landing zone and placed behind the mark of the vessel phantom and (**d, e**) the inserted balloon catheter over the wire with the mark of the vessel phantom located in its center.

The demonstrated MPI online reconstruction algorithm allowed real time tracking with about 2 volumes / second and at a spatial resolution of 2×2×1 mm^3^. Due to the real time imaging, an exact positioning of the magnetically labelled guidewire in front and behind the target mark on the vessel phantom was feasible as shown in Fig 5c and 5d. Additionally the angioplasty balloon catheter could be exactly placed with its two marks located equidistant to the target mark of the vessel phantom as shown in Fig 5e. See also S1 File, which demonstrates the Online-Reconstruction-Monitor showing online catheter positioning in three orthogonal planes.

### In-vitro angioplasty with a MPI-MRI road map approach

**Fig 6:**
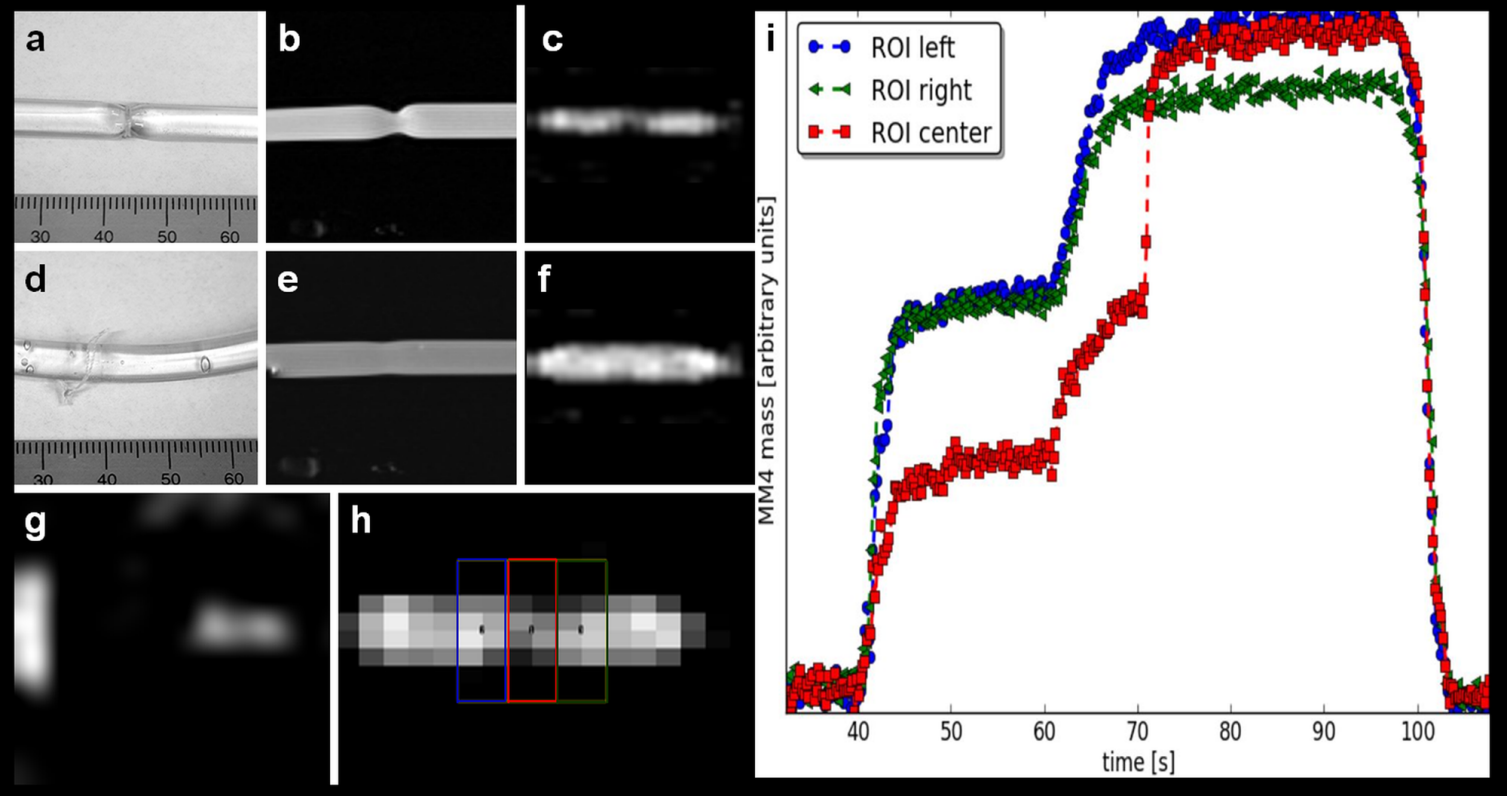
MPI-guided balloon angioplasty using MRI-MPI road map approach. (**a**) Photography of the vessel phantom with stenosis. (**b**) 2D PD-weighted MRI of the phantom with stenosis and angioplasty balloon inflated to 4.5 bar with MM4 for visualization **(c)** of the stenosis on sagittal MPI. **(d)** Photography of the vessel phantom after road map guided angioplasty with removal of the stenosis (ruptured string ligature). **(e)** MRI and **(f)** sagittal MPI verified the successful angioplasty. (**g**) MPI-MIP in z-projection of two of the three fiducials. **(h)** Four ROIs were placed over the balloon, light and dark blue outside the stenosis, the red and green within the stenosis. (**i**) The time curve during angioplasty showed the iron mass (arbitrary units); within the stenosis (red ROI) the iron mass is smaller at the beginning of dilatation but at 70s (20 bar) suddenly increases to the level of the blue and green colored ROIs indicating successful angioplasty.

Using the described setup of fiducial markers as shown in Figs 1a and 1b and 6g, it was feasible to identify all three fiducials of the phantom in both, MPI and MRI. Taking these three external landmarks together with the signal from the vessel phantom itself or the angioplasty balloon respectively a co-registration of MPI and MRI datasets was realized.

**Fig 7:**
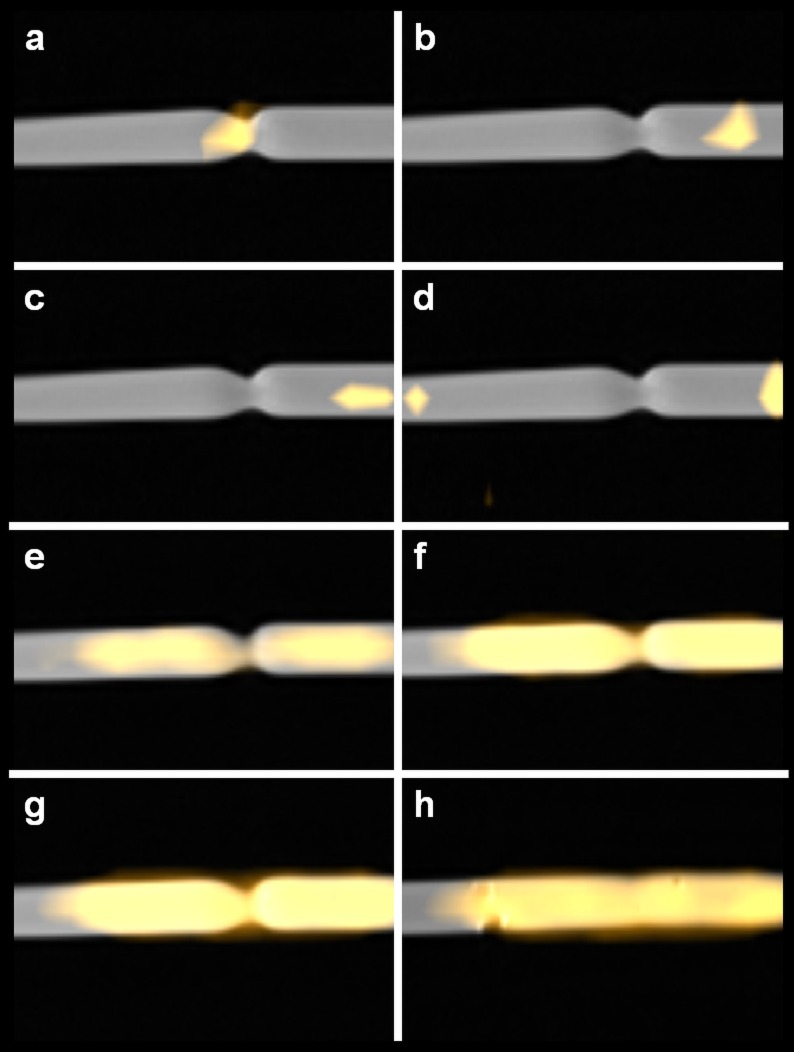
MPI/MRI road mapping of balloon angioplasty. MPI/MRI fused images in sagittal orientation (PD-weighted MRI and MPI-Signal) displayed in a timeline during the simulated angioplasty procedure. **(a)** The labeled guidewire was placed (from left to right) in the center and **(b, c)** beyond the stenosis. **(d)** The balloon catheter was placed over the wire passing the stenosis with two signals from the lacquer marks—compare Fig 3—barely fit within the FoV of the MPI. **(e)** The balloon catheter is slightly inflated to 4.5 bar adapting to the contour of the stenosis and thereby making it visible. **(f)** Then the balloon pressure is gradually increased and the balloon consecutively increases in diameter until **(g)** the ligature/string ruptures at a pressure of 20 bar simulating the removal of the vessel phantom stenosis. **(h)** Fused MPI-MRI image of the fully inflated balloon after successful *in-vitro* angioplasty, a minimal remaining stenosis is visible.

MR images were used for an anatomical road map and revealed a stenosis of 50% (Fig 7). Exact monitoring and positioning of the guidewire and angioplasty catheter within the stenosis was easy to achieve using the above-mentioned software and hardware setup. For details, see Tables 1–3. MRI Image fusion with real time MPI images as demonstrated in Fig 7 was feasible. Slight inflation of the balloon with SPIOs (MM4) allowed direct verification of the stenosis by MPI (Fig 6c). Fig 6h shows the placement of three regions of interests (ROIs) for monitoring the total iron mass (arbitrary units) over the time of angioplasty within the region of the stenosis (red) as well as before and after the stenosis (green and blue) (Fig 6i). At 40 sec., we began to inflate the balloon with MM4. When the pressure was held at 4.5 bar from 45 sec. till 60 sec. the difference of iron mass between the red ROI and the blue and green ROIs semi quantifies the extend of the stenosis. From 62 sec., the pressure was continuously increased up to 20 bar. After 72 sec., when the stenosis was removed, the signal of the red ROI matches the signal of the blue ROI. The smaller signal from within the green ROI from second 67 until 98 was due to a trapped air bubble. See S2 File, which demonstrates the placing of the angioplasty catheter and *in-vitro* angioplasty. After *in-vitro* angioplasty, MRI verified successful angioplasty (Fig 6e). The possibility of real time MPI allowed for exact positioning of the instruments and documents the use of this approach for MPI-MRI-guided *in-vitro* intervention and angioplasty.

### In-vitro angioplasty with MPI guidance and a blood pool tracer approach

**Fig 8:**
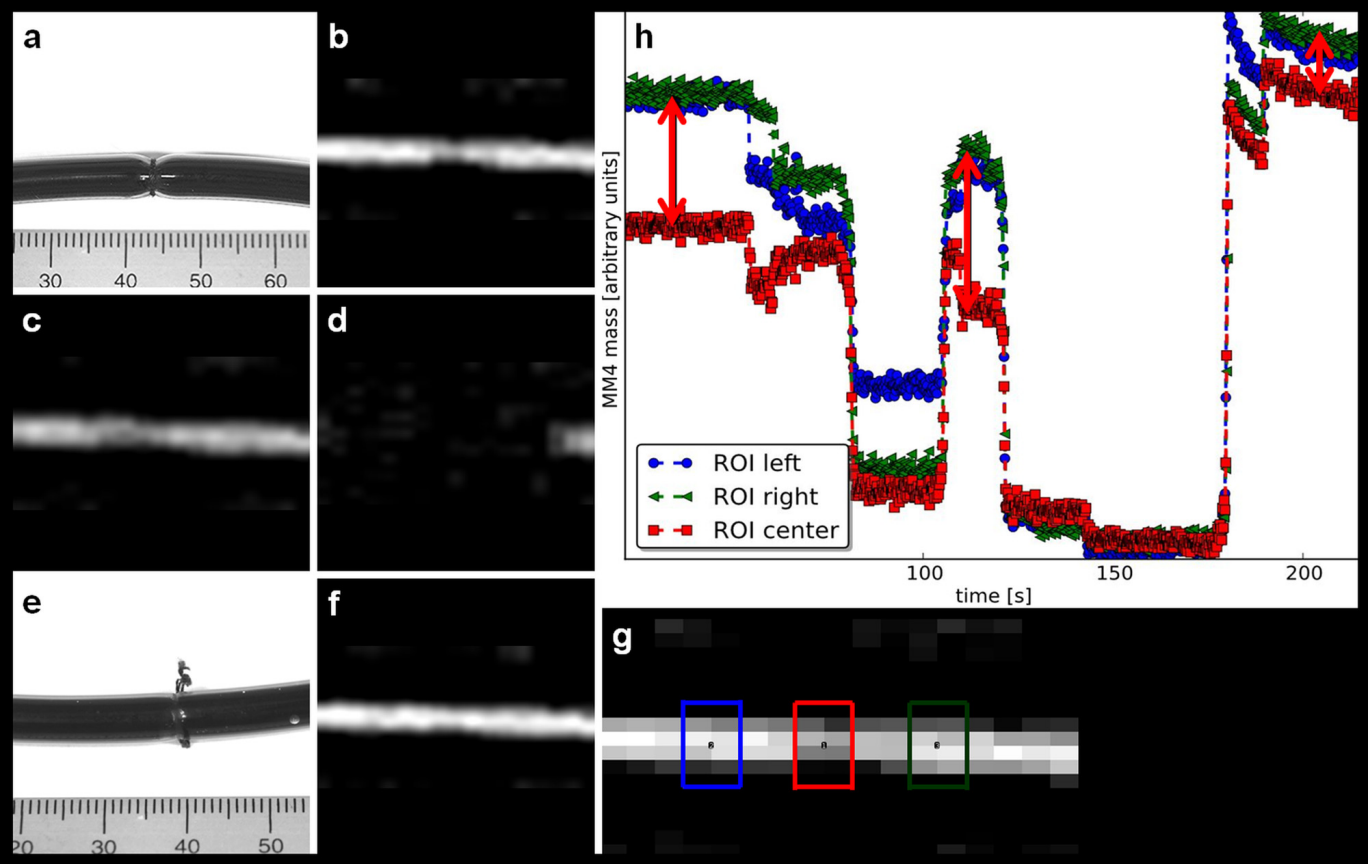
MPI-guided balloon angioplasty using blood pool contrast agent approach. **(a)** Photography of a MM4 filled vessel phantom with the central stenosis and **(b)** corresponding MPI-MIP slice in y-projection. **(c)** The MM4 is partially displaced when the angioplasty balloon is placed within the region of the stenosis and **(d)** fully displaced during balloon inflation with saline to a maximum of 20 bar. When the angioplasty balloon is removed and the region of the stenosis is refilled with MM4, the success of the angioplasty is verified **(e)** by photography and (**f**) MPI. (**g**) Two ROIs were placed outside the stenosis (red and blue) and one (green) in the center of the stenosis. (**h**) The quantitative analysis showed the iron mass (arbitrary units) during the angioplasty procedure (the difference of the SPIOs amount (iron mass) describes the degree of the vessel phantom stenosis indicated by lengths of arrow line). After intervention the difference of iron mass between all three ROIs is decreased, indicating the success of the angioplasty.

The stenosis of the MM4 filled vessel model (Fig 8a) was detected by the lower signal in the region of the stenosis due to less amounts of MM4 present and therefore less iron mass (Fig 8b). For monitoring the iron mass over time, three ROIs were placed within (“red”) as well as equidistant (“blue” and “green”) to the stenosis (Fig 8g). The initial signal differences of the central “red” ROI within the stenosis compared to the signal of the “green” and “blue” ROI outside the stenosis semi quantifies the degree of narrowing (Fig 8h). When the balloon catheter was slightly inflated with saline to 3.0 bar and reached the region of the stenosis, a signal loss is visible due to the MM4 displacement allowing indirect monitoring of the instruments’ position (Fig 8c and 8h, second 70 to 105). When initial positioning was performed (Fig 8h, second 80 to 110), the signal within the “blue” ROI did not decrease to the level of the “green” ROI, indicating that the balloon was placed excentrically to the stenosis. When the catheter is retrieved and MM4 fills the region again, the signal difference between the “red” ROI and the “blue” and “green” ROI the stenosis is still present (Fig 8h, second 105 to 120). After correct replacement with a signal drop in all three ROIs (Fig 8h, second 125 to 145), the catheter was fully inflated to 20 bar and all MM4 in the region of the balloon displaced (Fig 8h, second 150 to 175). When retrieved again, the stenosis was no longer visible on sagittal MPI (Fig 8f). The detected signal difference in the three ROIs is only marginal, indicating successful intervention (Fig 8h, second 155 to 220) and is caused by a minimal remaining stenosis compare (Fig 7h).

## Discussion

The basis for cardiovascular interventional MPI was set by Haegele et al. in 2012. They presented the feasibility of MPI of SPIOs labeled endovascular instruments at high temporal resolution [6] and introduced the first magnetic coating of interventional devices for MPI visualization with a coating thickness of about 500 μm [13]. Various interventional instruments have been investigated concerning potential heating in MPI [7]. For our study we used instruments that showed neither heating nor artifacts in MPI.

A key feature for interventional MPI is the possibility of real time visualization [14]. 4D intervention has been demonstrated on bases of a gantry-based x-ray flat detector system [15]. The authors emphasized that real time 4D imaging will make vascular interventions safer and faster and would facilitate more complex interventional approaches [16]. We emphasize the same for 4D MPI with the additional advantage of a radiation free imaging modality. The possibility of real time 4D MPI acquisition was introduced in 2009 [3], but real time visualization was not established until now. Knopp et al. presented several solutions to increase speed of data reconstruction such as model-based reconstruction [17], weighted iterative reconstruction [12] and sparse reconstruction algorithms [18]. In our study the iterative reconstruction algorithm [12] used to reconstruct 3D MPI data [3, 9] was implemented to enable for reconstruction of about 2 volumes per second using a reconstruction grid of size 25^3^. Advanced matrix compression techniques [19, 20] can speed up reconstruction times by several orders but these techniques can yield artifacts we wanted to avoid. Displaying 2 volumes per second proved to be sufficient for real time 4D MPI tracking of endovascular devices.

The thin layer of fixed contrast agent (magnetic lacquer) for labeling the devices, enabled for coaxial use of the guide wire. The study presents two approaches for MPI-guided angioplasty, first an MPI/MRI roadmapping and secondly an MPI-guided blood pool tracer approach.

Kaul et al. recently presented a workflow for combined MP/MR imaging [21]. Using the same approach, the presented MPI/MRI roadmapping approach combines highly space-resolved anatomic MR imaging with high temporal-resolved and sensitive 4D MP imaging. In this study the roadmapping was performed using a 7T MRI located right next to the MPI scanner to avoid longer transportation of the experimental setup between the MRI and MPI scanner. In principle all common clinical MRI-Systems at lower field strengths could be easily used to perform the roadmapping in this experimental setting as well as in a possible future clinical setup. Magnetic labeling of endovascular instruments but no SPIO application was needed to perform intervention. This approach avoids SPIO accumulation in the reticuloendothelial system (RES) of liver and spleen [22], allowing theoretically unlimited repetitions of interventions. In an *in-vivo* setting in humans the MPI/MRI image fusion might be difficult to realize, e.g. in small vessels of peripheral body regions due to motion artifacts (e.g. breathing, cardiac motion of bowel movement). Bimodal external fiducial markers, in principle similar to those used in this study, emerge as a possible solution.

Second, the contrast agent approach delivered a positive vessel contrast enabling for MPI-based diagnostic and intervention. So far navigation of labeled devices within a SPIOs filled vessel phantom is challenging since localization of the device is only possible indirectly due to displacement of the tracer. To distinguish the MPI signal from the device and surrounding tracer (e.g. blood pool SPIOs [23]), the technique of multi spectral, or also named “colored MPI” offers an interesting approach [24, 25]. SPIOs of the blood circulation can in principle be distinguished from a fixed contrast agent on the catheter material and visualized in different colors. The formerly clinically used contrast agent ferucarbotran (Resovist^®^) can only be used as a bolus tracer [26]. There is no MPI blood pool tracer with renal clearance, which could lead a way out of the dilemma of potentially overloading the RES with iron and allowing for multiple injections. With future increasing sensitivity of MPI systems, e.g. by using a field free line [27, 28] or with availability of more potent SPIO tracers these obstacles might be overcome.

The major limitation of this study is the inherent character of a proof of principle study. The used magnetic lacquer is not promptly applicable in an *in-vivo* setting. To enable future *in-vivo* studies further investigations are needed to establish a biocompatible magnetic coating for endovascular devices or integrate the tracer into the devices’ polymer. A further limitation is the considerably small field of view of the MPI scanner used in this preclinical study. A recent study presents the possibility of joint reconstruction of non-overlapping focus fields [29], giving the bases for future investigations on dynamic focus fields. In the last years open scanner designs with possible advantages for interventional MPI [30] have been discussed with ongoing investigations on realizing the first human scaled whole body MPI scanner [31]. For future scanner designs a combination of MPI with an anatomical image modality such as MRI is being investigated [32].

Despite those limitations, the presented study provides the proof of principle that magnetic particle imaging allows for 4D real time tracking of interventional devices, guided positioning, balloon angioplasty and MRI based roadmapping. Using the first commercial MPI scanner the next step towards radiation free interventional MPI is demonstrated.

## Supporting Information

S1 File Online Reconstruction Monitor. Video of the Online-Reconstruction-Monitor in four times speed showing online catheter positioning in three orthogonal planes. Real time signal intensity is displayed in arbitrary units (left bottom). Images taken with 46 frames/s.
(WMV)

S2 File Road Map angioplasty. Video of a Road Map angioplasty displayed at five times speed. Fusion video of static sagittal PDw MRI Road Map and MPI overlay showing the balloon inflation with MM4 for in-vitro angioplasty.
(WMV)
